# Cardiac Shock Wave Therapy for Coronary Heart Disease: an Updated Meta-analysis

**DOI:** 10.21470/1678-9741-2019-0276

**Published:** 2020

**Authors:** Hai-Tao Yang, Xiang Xie, Xian-Geng Hou, Wen-Juan Xiu, Ting-Ting Wu

**Affiliations:** 1 Department of Cardiology, First Affiliated Hospital of Xinjiang Medical University, Urumchi, People’s Republic of China.; 2 Department of Cardiology, Changji Hui Autonomous Prefecture People’s Hospital, People’s Republic of China.

**Keywords:** Extracorporeal Shockwave Therapy, Exercise Test, Coronary Disease, Heart, Walking, Confidence Intervals

## Abstract

**Introduction:**

The aim of this article is to study the efficacy and safety of cardiac shock wave therapy (CSWT) in the treatment of coronary heart disease (CAD).

**Methods:**

A comprehensive search of electronic databases and a manual search of conference papers and abstracts were performed until September 30, 2018. The studies using RevMan 5.3 and STATA 14.0 softwares were reviewed, and meta-analyses were performed on 13 indicators, such as a six-min walking distance test (6MWT), New York Heart Association (NYHA) functional class, Seattle Angina Questionnaire (SAQ) score, angina class (Canadian Cardiology Society [CCS]), etc.

**Results:**

A total of 26 articles were included. The total patient population was 855, of which 781 patients were treated with CSWT. Meta-analyses indicated that 6MWT (mean difference [MD] 75.64, 95% confidence interval [CI] 49.03, 102.25, *P*<0.00001) and NYHA (MD -0.70, 95% CI -0.92) in the CSWT group were comparable to those in the conventional revascularization group (MD -0.70, 95% CI -0.92, -0.49, *P*<0.00001). SAQ (MD 10.75, 95% CI 6.66, 14.83, *P*<0.00001), CCS (MD -0.99, 95% CI -1.13, -0.84, *P*<0.00001), nitrate dosage (MD -1.84, 95% CI -2.77, -1.12, *P*<0.00001), LVEF (MD 3.77, 95% CI 2.17, 5.37, *P*<0.00001), and SSS (MD -4.29, 95% CI -5.61, -2.96, *P*<0.00001), SRS (MD -2.90, 95% CI -4.85, -0.95, *P*=0.004), and the exercise test (standard mean difference 0.57, 95% CI 0.12, 1.02, *P*=0.01) all showed significant differences.

**Conclusion:**

CSWT may offer beneficial effects to patients with CAD, but more large-scale clinical studies are needed to further verify its therapeutic effect.

**Table:** 

Abbreviations, acronyms & symbols				
**ACEI**	**= Angiotensin converting enzyme inhibitors**		**NO **	**= Nitric oxide**
**ARB**	**= Angiotensin-receptor blockers**		**NR**	**= No report**
**BMI**	**= Body mass index**		**NYHA**	**= New York Heart Association**
**CABG**	**= Coronary artery bypass grafting**		**PCI**	**= Percutaneous coronary intervention**
**CAD**	**= Coronary heart disease**		**PICOS**	**= Participants, interventions, comparisons, outcomes, and study design**
**CCB**	**= Calcium channel blockers**		**PRISMA**	**= Preferred Reporting Items for Systematic Reviews and Meta-Analyses**
**CCS**	**= Canadian Cardiology Society**		**RCT**	**= Randomised controlled trial**
**CI**	**= Confidence interval**		**SAQ**	**= Seattle Angina Questionnaire**
**CSWT**	**= Cardiac shock wave therapy**		**SD**	**= Standard deviation**
**EMBASE**	**= Excerpta Medica dataBASE**		**SDS**	**= Total difference score**
**IV**	**= Inverse variance methods**		**SE**	**= Standard error**
**LVEDD**	**= Left ventricular end-diastolic diameter**		**SMD**	**= Standard mean difference**
**LVEDV**	**= Left ventricular end-diastolic volume**		**SPECT**	**= Single-photon emission computed tomography**
**LVEF**	**= Left ventricular ejection fraction**		**SRS**	**= Total resting score**
**LVESV**	**= Left ventricular end-systolic volume**		**SSS**	**= Total load score**
**MD**	**= Mean difference**		**VEGF**	**= Vascular endothelial growth factor**
**MEDLINE**	**= Medical Literature Analysis and Retrieval System Online**			
**6MWT**	**= Six-min walking distance test**			

## INTRODUCTION

Coronary heart disease (CAD) refers to coronary atherosclerosis, which causes vascular stenosis or occlusion and further leads to heart disease caused by myocardial ischaemia, hypoxia, or necrosis. It caused seven million deaths worldwide in 2010^[1]^. The current treatment methods are based on drug therapy, percutaneous coronary intervention (PCI), or coronary artery bypass grafting (CABG). Although PCI or CABG can reopen the blood vessels of the patients or ensure the blood flow of the main blood vessels, these methods cannot eliminate microvascular occlusion, paralysis, or loss. At the same time, for severe CAD, cardiac dysfunction is often caused by long-term coronary artery multivessel disease, resulting in a large area of myocardial cell necrosis, fibrosis, and decreased ventricular compliance. Part of the population lacks the indication for PCI or CABG or has clinical prognoses that are poor. In this context, cardiac shock wave therapy (CSWT) has become a new mean of improving heart disease treatment. CSWT is the latest development of cutting-edge technology in the world, and it has created a new concept and method of CAD treatment. This technology was developed by Switzerland and Germany. It passed the EC certification as early as 2004. It is widely used in many European countries such as Switzerland, Germany, and Italy, and in Asia. China also provides reports every year. CSWT is a non-invasive treatment for CAD. Its mechanism of action is to produce mechanical shear and cavitation effects in myocardial tissue cells, thereby producing nitric oxide (NO) and vascular endothelial growth factor (VEGF) in the local myocardium. VEGF promotes microvascular regeneration, improves myocardial blood supply, and reduces cardiac ischaemic events^[2,3]^. Currently, there are quite a few clinical trials that have reported the clinical effects of CSWT. In 2018, a multi-centre study by Yoku^[4]^ examined 41 patients with ischaemic cardiomyopathy and included three months of follow-up before and after treatment. The evaluation of symptoms, exercise tolerance, cardiac function, and other indicators confirmed the effectiveness and safety of CSWT. However, in the article, the data provided by each centre are too small, and some indicators are skewed and cannot represent the general population. In the existing study, the clinical prognosis evaluation differs greatly due to the small sample size of the general clinical study. Evgeny et al.^[5]^ conducted a randomized controlled trial (RCT) of 72 patients and assessed exercise tolerance and found no significant differences between the two groups. Prior to this, the reports of improvement in exercise tolerance in the study by Prasad et al.^[6]^ and Cassar et al.^[7]^ were significant. Therefore, this study systematically illustrates the effects of CSWT by performing a meta-analysis on existing clinical research data.

## METHODS

### Search Strategy

Comprehensive literature searches of major electronic databases (PubMed, Medical Literature Analysis and Retrieval System Online [MEDLINE], Excerpta Medica database [EMBASE], Elsevier, and Google Scholar) were performed. The keywords we searched for were “extracorporeal cardiac shock wave therapy”, “myocardial shock wave therapy”, “CSWT”, “ESWT” plus “coronary artery disease”, “ischaemic heart disease”, “refractory angina treatment”, “stable”, and “angina treatment". The search deadline was September 30, 2018. In addition, we manually searched conference papers and conference abstracts of the American College of Cardiology, American Heart Association, and European Society of Cardiology.

### Inclusion and Exclusion Criteria

Inclusion criteria were as follows: (1) study with randomized controlled or single-arm or cohort study design; (2) patients with clear diagnosis of CAD; (3) experimental design in the experimental group for the treatment of CAD based on conventional CSWT treatment (shock energy of 0.09 mJ/mm^2^), the control group for CAD drug treatment; (4) prognostic indicators: clinical endpoints such as six-min walking distance test (6MWT), New York Heart Association (NYHA), total load score (SSS), and left ventricular ejection fraction (LVEF). Exclusion criteria were as follows: (1) animal testing; (2) non-English literature; (3) experimental group for low-energy CSWT treatment.

### Screening Literature and Data Extraction

**Fig. 1:**
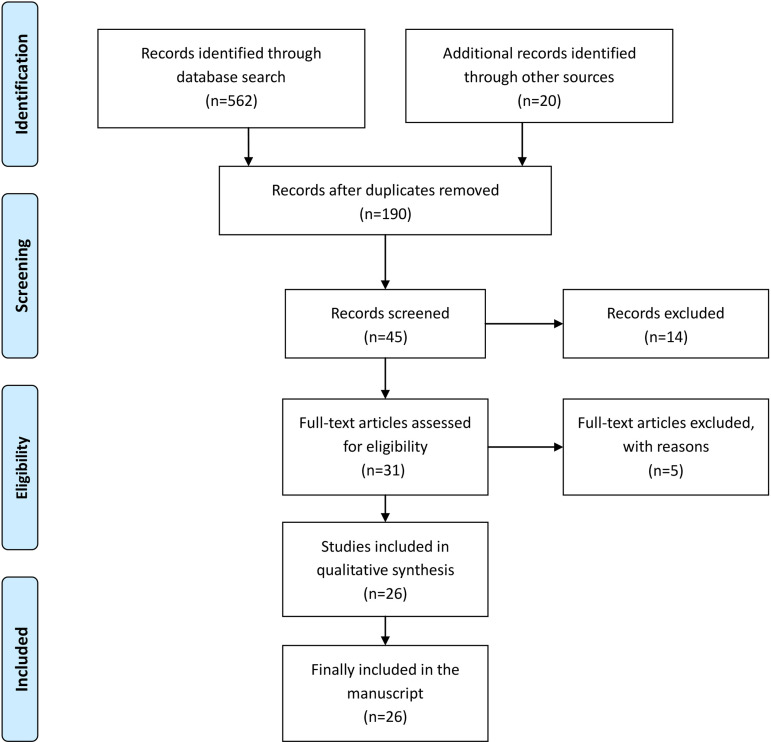
The study flow diagram.

The literature was independently reviewed by two researchers (YHT and XWJ) and it was considered or not based on the inclusion and exclusion criteria, following the Preferred Reporting Items for Systematic Reviews and Meta-Analyses (PRISMA) statement^[8]^ (Figure 1, Table 1). If the researchers disagreed, a third researcher (XX) joined for brainstorming and further decided to withdraw. After the first search and summary, the preliminary screening was carried out by reading the title and abstract of the literature. The second screening included a further reading of the literature, and the data were extracted from it. If the literature did not involve observation indicators, the first author was contacted. The literature screening process is shown in Figure 1. The literature included 26 studies that met the criteria. Literature data extraction includes the author, publication period, number of study samples, type of study, follow-up time, and patient age, male ratio, body mass index, and research population characteristics. The literature data extraction results are shown in Table 2.

**Table 1:** The Preferred Reporting Items for Systematic Reviews and Meta-Analyses (PRISMA) statement^[8]^.

Section/topic		#	Checklist item	Reported on page #
**Title**	Title	1	Identify the report as a systematic review, meta-analysis, or both.	√
**Abstract**	Structured summary	2	Provide a structured summary including, as applicable: background; objectives; data sources; study eligibility criteria, participants, and interventions; study appraisal and synthesis methods; results; limitations; conclusions, and implications of key findings; systematic review registration number.	√
**Introduction**	Rationale	3	Describe the rationale for the review in the context of what is already known.	√
	Objectives	4	Provide an explicit statement of questions being addressed with reference to participants, interventions, comparisons, outcomes, and study design (PICOS).	√
**Methods**	Protocol and registration	5	Indicate if a review protocol exists, if and where it can be accessed (*e.g*., Web address), and, if available, provide registration information including registration number.	none
	Eligibility criteria	6	Specify study characteristics (*e.g*., PICOS, length of follow-up) and report characteristics (*e.g*., years considered, language, publication status) used as criteria for eligibility, giving rationale.	√
	Information sources	7	Describe all information sources (*e.g*., databases with dates of coverage, contact with study authors to identify additional studies) in the search and date last searched.	√
	Search	8	Present full electronic search strategy for at least one database, including any limits used, such that it could be repeated.	√
	Study selection	9	State the process for selecting studies (*i.e*., screening, eligibility, included in systematic review, and, if applicable, included in the meta-analysis).	√
	Data collection process	10	Describe method of data extraction from reports (*e.g*., piloted forms, independently, in duplicate) and any processes for obtaining and confirming data from investigators.	√
	Data items	11	List and define all variables for which data were sought (*e.g*, PICOS, funding sources) and any assumptions and simplifications made.	√
	Risk of bias in individual studies	12	Describe methods used for assessing risk of bias of individual studies (including specification of whether this was done at the study or outcome level), and how this information is to be used in any data synthesis.	√
	Summary measures	13	State the principal summary measures (*e.g*., risk ratio, difference in means).	√
	Synthesis of results	14	Describe the methods of handling data and combining results of studies, if done, including measures of consistency (e.g., I^2^) for each meta-analysis.	√
	Risk of bias across studies	15	Specify any assessment of risk of bias that may affect the cumulative evidence (*e.g*., publication bias, selective reporting within studies).	√
	Additional analyses	16	Describe methods of additional analyses (*e.g*., sensitivity or subgroup analyses, meta-regression), if done, indicating which were pre-specified.	√
**Results**	Study selection	17	Give numbers of studies screened, assessed for eligibility, and included in the review, with reasons for exclusions at each stage, ideally with a flow diagram.	√
	Study characteristics	18	For each study, present characteristics for which data were extracted (*e.g*., study size, PICOS, follow-up period) and provide the citations.	√
	Risk of bias within studies	19	Present data on risk of bias of each study and, if available, any outcome level assessment (see Item 12).	√
	Results of individual studies	20	For all outcomes considered (benefits or harms), present, for each study: (a) simple summary data for each intervention group (b) effect estimates and confidence intervals, ideally with a forest plot.	√
	Synthesis of results	21	Present results of each meta-analysis done, including confidence intervals and measures of consistency.	√
	Risk of bias across studies	22	Present results of any assessment of risk of bias across studies (see Item 15).	√
	Additional analysis	23	Give results of additional analyses, if done (*e.g*., sensitivity or subgroup analyses, meta-regression [see Item 16]).	√
**Discussion**	Summary of evidence	24	Summarize the main findings including the strength of evidence for each main outcome; consider their relevance to key groups (*e.g*., healthcare providers, users, and policy makers).	√
	Limitations	25	Discuss limitations at study and outcome level (*e.g*., risk of bias), and at review-level (*e.g*., incomplete retrieval of identified research, reporting bias).	√
	Conclusions	26	Provide a general interpretation of the results in the context of other evidence, and implications for future research.	√
**Funding**	Funding	27	Describe sources of funding for the systematic review and other support (*e.g*., supply of data); role of funders for the systematic review.	√

From: Moher D, Liberati A, Tetzlaff J, Altman DG, The PRISMA Group (2009). Preferred Reporting Items for Systematic Reviews and Meta-Analyses: The PRISMA Statement. PLoS Med 6(6): e1000097. doi:10.1371/journal.pmed1000097 For more information, visit: www.prisma-statement.org.

**Table 2:** Literature baseline data extraction table.

Trials	Hong Yan CAI^[19]^ (2015)	Megha^[6]^ (2015)	Gianluca^[20]^ (2015)	M. Kaller^[18]^ (2015)	S. Nirala^[15]^ (2016)	J. Vainer^[16]^ (2016)	Wenxia Wang^[14]^ (2016)	J. Slikkerveer^[17]^(2016)	Gianluca Alunni^[13]^ (2017)	Masahiro Myojo^[12]^ (2017)	Evgeny^[5]^ (2018)	Anderson S^[9]^ (2018)	Massimo Slavich^[11]^ (2018)
Patients (n)	26	111	72	21	52	33	23	15	72	6	72	19	23
CSWT/placebo (n)	26	111	43/29	21	41/11	33	23	15	72	6	37/35	19	19/4
Study design	Single-arm study	Single-arm study	Cohort study	Single-arm study	Cohort study	Single-arm study	Single-arm study	Single-arm study	Single-arm study	Single-arm study	RCT	Single-arm study	Cohort study
Age (years)	63±10	62.9±10.9	70±5.3/ 71±5.3	65±10	63.4±10.8/ 71±6.52	69.7±8	67±6	NR	74.6±14.7	NR	67.6±8.3/ 68.8±8.3	64±13	69.79±10.22/ 65.25±5.74
Male (%)	88.46	83.7	83.7/79	61.9	35/8	82	73.91	NR	79	83.3	62.3/82.3	74	79/75
BMI	23.86	23.9±6.0	NR	NR	23.9±2.7/ 23.21±2.35	29±4.6	NR	NR	NR	NR	29.7±4.1/ 30.1±3.8	NR	NR
LVEF	NR	NR	50.4±10.3/ 57.3±9.6	NR	NR	55±12%	NR	51.8±15.2	56±12	NR	54.5±9.1/ 56.5±7.1	52.4±10.4	NR
**Cardiovascular risk factors**													
Smoking (%)	NR	45	NR	NR	36.54	24	NR	NR	11	NR	5.4/17.1	37	73%/50%
Hypertension (%)	73.08	77.4	100/100	80.9	NR	76	NR	NR	98	50	96.3/97.1	95	79%/100%
Diabetes (%)	26.92	51.4	32.5/27	52.3	30.77	46	NR	NR	35	33.3	21.6/28.8	63	42%/75%
Dyslipidaemias (%)	15.38	86	95.3/96	NR	1.92	94	NR	NR	94	83.3	83.8/85.7	95	79%/100%
**Condition**													
Previous PCI (%)	3.85	29.4	88.4/72	76.1	61.54	55	NR	25	80	100	51.4/82.9	NR	NR
Previous CABG (%)	3.85	31.2	48.8/31	90.4	NR	76	NR	75	42	33.3	54.1/57.1	NR	NR
Previous myocardial infarction	NR	48.9	32.5/27	52.3	NR	NR	NR	NR	NR	50	51.4/82.9	NR	48
**Medical therapy**													
Aspirin (%)	NR	96	93/96	66.6	NR	64	NR	NR	NR	50	NR	NR	NR
Clopidogrel (%)	NR	NR	41.8/37	52.3	NR	30	NR	NR	NR	33.3	NR	47	NR
ACEI/ARB (%)	NR	66.7	NR	90.4	NR	36/21	NR	NR	54(ACEI)/ 43 (ARB)	86.6	NR	79	NR
b-blockers (%)	NR	91.9	90/89	100	NR	85	NR	NR	90	86.6	NR	100	84/100
CCB (%)	NR	60.8	NR	19.4	NR	79	NR	NR	60	33.3	NR	NR	32/50
Statin (%)	NR	NR	90/93	90.4	NR	NR	NR	NR	84	100	NR	100	NR
Diuretic (%)	NR	NR	NR	61.9	NR	NR	NR	NR	NR	33.3	NR	NR	NR
Nitrates (%)	NR	88.6	72/69	100	NR	91	NR	NR	73	33.3	NR	89	53/75
Patients (n)	42	25	9	10	25	24	24	16	9	20	86	55	25
CSWT/placebo (n)	42	25	9	10	25	24	24	16	9	20	43/43	20/14	14/11
Study design	Single-arm study	Single-arm study	Single-arm study	Single-arm study	Single-arm study	Single-arm study	Single-arm study	Single-arm study	Single-arm study	Single-arm study	Control study	RCT	RCT
Age (years)	71.0±12.2	66±7.3	66.78±8.83	NR	63.8±8.2	63.8±8.2	63±6.1	66±10	5.11±5.46	69.45±8.73	58.7±9.5/ 56.6±11.6	62.7±12.0/ 67.9±7.8	63.7±8.60/ 66.45± 8.51
Male (%)	80.9	NR	55.6	NR	NR	75	83.3	NR	100	80	87/84	90/85.7	71.4/72.7
BMI	25.0±3.7	NR	NR	NR	NR	30±4.6	NR	NR	NR	NR	NR	24.5±2.8/ 24.0±3.2	NR
LVEF	55.6±15.3	NR	NR	NR	NR	NR	32.2±6.0	NR	NR	51.45%±8.03	49.6±14.1/ 43.6±15.7	NR	NR
**Cardiovascular risk factors**													
Smoking (%)	5	NR	0	NR	NR	29.1	NR	NR	NR	NR	NR	35.0/42.9	50.0/54.5
Hypertension (%)	79	NR	55.6	NR	NR	100	NR	NR	88.8	73.08	56/44	65/71.4	57.1/45.5
Diabetes (%)	40	NR	66.7	NR	NR	37.5	NR	NR	55.5	26.92	77/74	35/28.6	42.9/45.5
Dyslipidaemias (%)	62	NR	44.4	NR	NR	100	NR	NR	33.3	15.38	NR	20/42.9	64.3/63.6
**Condition**													
Previous PCI (%)	40	NR	33.3	NR	NR	83.3	2	NR	NR	3.85	NR	NR	NR
Previous CABG (%)	14	NR	44.4	NR	NR	79.1	NR	NR	NR	3.85	NR	NR	NR
Previous myocardial infarction	52	NR	NR	NR	NR	45.8	50	NR	NR	NR	NR	65/57.1	NR
**Medical therapy**													
Aspirin (%)	90	NR	77.8	NR	NR	NR	91.7	NR	100	90	NR	85/85.7	71.4/63.6
Clopidogrel (%)	NR	NR	NR	NR	NR	83.3	NR	NR	NR	35	NR	NR	NR
ACEI/ARB (%)	NR	NR	55.6	NR	NR	70.8	100	NR	77.7	18/5	NR	NR	64.3/72.7
b-blockers (%)	93	NR	88.9	NR	NR	100	95.8	NR	77.7	95	NR	90/92.9	64.3/63.6
CCB (%)	NR	NR	88.9	NR	NR	45.8	NR	NR	22.2	30	NR	30/28.6	63.6/45.5
Statin (%)	81	NR	44.4	NR	NR	100	54.2	NR	88.8	85	NR	90/92.9	64.3/81.8
Diuretic (%)	NR	NR	NR	NR	NR	NR	58.3	NR	33.3	35	NR	15/14.3	35.7/36.4
Nitrates (%)	67	NR	55.6	NR	NR	75	62.5	NR	22.2	100	NR	40/28.6	64.3/54.5

ACEI=angiotensin converting enzyme inhibitors; ARB=angiotensin-receptor blockers; CABG=coronary artery bypass grafting; CCB=calcium channel blockers; CSWT=cardiac shock wave therapy; BMI=body mass index; LVEF=left ventricular ejection fraction; NR=no report; PCI=percutaneous coronary intervention; RCT=randomised controlled trial

### Statistical Analysis

This study used the softwares RevMan, version 5.3, and STATA, version 14.0. Due to the non-normal distribution of some of the included studies, data were indicated by the median and interquartile range. Because the sample size is small, if the original data were not obtained after contacting the first author, the relevant data were eliminated. The data in this study are measurement data, and the mean difference (MD) and 95% confidence interval (CI) are used as the effective amounts for the measurement data. The heterogeneity analysis included in the study was performed by a Q test. The heterogeneity was expressed by I^2^ value and *P*-value. If the *P*-value was > 0.1 and the I^2^ value was < 50%, the fixed effect model was used for the meta-analysis; if neither of the conditions met, a random effects model was used. When the research unit is unified in the research data, the effect indicator selects the MD mode. If the research unit is not uniform, the effect indicator selects the standard mean difference (SMD) mode. In the data analysis, the indicators of heterogeneity are further explored to find the cause of heterogeneity. Subgroup analysis and sensitivity analysis for the reasons of possibility of heterogeneity and indexing of the indicators were done to judge publication bias.

## RESULTS

### Literature Search Results

**Fig. 2:**
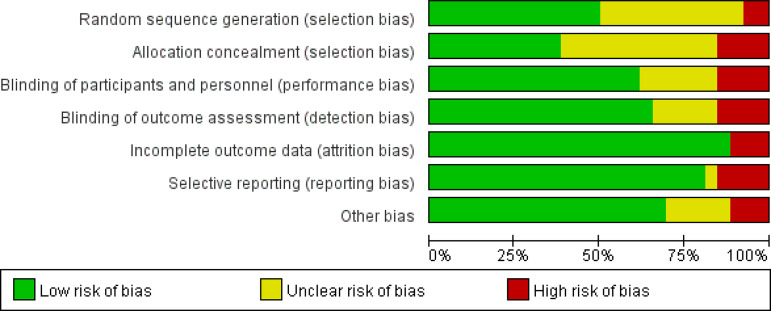
Document quality evaluation.

This study included 26 clinical studies^[5-7,9-34]^ and a total of 855 patients with CAD, including 781 patients treated with CSWT and 74 patients receiving drug treatment. This study included the follow-up time span from the literature. The shortest time of follow-up was for CSWT. The longest observation period was 72 months. After analysing the data in the sample study, the data were summarized and five documents were excluded because there were no unified data. The study analysed 26 data sets from the literature. Document quality evaluation is shown in Figure 2. The final observation indexes of the study were as follows: (1) 6MWT; (2) heart failure class (NYHA functional class); (3) Seattle Angina Questionnaire (SAQ) score; (4) angina class (Canadian Cardiology Society [CCS]); (5) nitrate dosage; (6) LVEF; (7) left ventricular end-diastolic volume (LVEDV); (8) left ventricular end-systolic volume (LVESV); (9) left ventricular end-diastolic diameter (LVEDD); (10) SSS; (11) total resting score (SRS); (12) total difference score (SDS); and (13) exercise test; the data extraction results can be seen in Table 3. SSS, SRS, and SDS are semiquantitative indicators for performing load-resting myocardial perfusion single-photon emission computed tomography (SPECT) examination. It divides the myocardium into fixed segments, each segment uses a four-point scale to evaluate the perfusion image. The resting image scores of each segment are summed to obtain SRS and the load image scores for each segment are added to the SSS. The difference between the two is the SDS.

**Table 3:** Original document endpoint data.

	6MWT	NYHA	SAQ	CCS	Nitrate dosage	LVEF	LVEDV	LVESV	LVEDD	SSS	SRS	Exercise test	SDS
Gianluca (2015)	NR	1.73±0.59 1.23±0.42	NR	1.92±0.69 1.33±0.57	NR	56±9 55±9	NR	NR	NR	21.2±10.3 14.3±10	13.4±9.28 10±9.8	NR	NR
M. Kaller (2015)	NR	NR	NR	NR	NR	54±17 51±14	91±34 96±25	47±33 49±24	55±7 53±6	NR	NR	NR	NR
Yu Wang (2010)	296±123.2 362±97.65	2.44±0.68 1.78±1.03	66±9.07 72.78±8.16	2.89±0.87 2.11±0.99	2.22±1.47 1.22±1.23	NR	NR	NR	NR	NR	NR	NR	NR
Waqar H (2012)	NR	2.48±0.6 1.95±0.5	NR	2.63±0.7 1.95±0.8	NR	42.6±15.6 52.7±10.4	NR	NR	NR	NR	NR	10.1±4.2 20.1±15.7	NR
Yu Wang (2012)	348.43±132.06 477.95±105.34	NR	59.21±15.66 79.63±9.87	NR	NR	NR	NR	NR	NR	NR	NR	NR	NR
PingYang (2013)	NR	NR	NR	NR	NR	48.60±4.48 56.42±4.61	NR	NR	56.00±3.85 51.21±3.79	NR	NR	NR	NR
			
Christoph K (2008)	NR	NR	NR	3.2±0.08 2.2±0.1	NR	NR	NR	NR	NR	NR	NR	NR	NR
Yury A (2010)	414±141 538±116	2.2±0.8 1.7±0.7	NR	2.6±0.7 1.9±0.7	NR	32.2±6.0 37.7±9.5	NR	NR	NR	28.2±8.4 24.6±6.4	23.9±8.1 21.4±7.1	NR	NR
Megha (2015)	NR	NR	NR	NR	1.14±1.01 0.52±0.68	NR	NR	NR	NR	26.49±19.38 23.88±19.90	16.62±17.77 15.82±15.28	457.0±146.8 606.0±126.4	9.53±17.87 7.77±11.83
Ahmed A (2007)	NR	NR	NR	3.3± 0.5 1.0±1.3	NR	NR	NR	NR	NR	8.3 ± 2.2 3.0 ± 8.1	NR	NR	NR
Yoshihiro (2005)	NR	NR	NR	2.7±0.2 1.8±0.2	5.4±2.5 0.3±0.3	NR	NR	NR	NR	NR	NR	NR	NR
Masahiro Myojo (2017)	NR	NR	NR	NR	NR	53.4±15.3 51.2±12.8	NR	NR	49.4±3.4 48.4±3.6	NR	NR	NR	NR
S. Nirala (2016)	388.90±83.04 445.80±172.41	2.09±0.94 1.04±0.49	72.72±12.33 79.92±25.14	2.18±0.75 1.14±0.57	0.21±0.82 2.00±1.18	NR	NR	NR	NR	NR	NR	NR	NR
J. Vainer (2016)	NR	NR	NR	3.0±0.3 1.7±0.7	9.7±7.0 1.5±1.9	53.2±12.3 53.7±12.1	NR	NR	NR	NR	NR	7.4±2.8 8.8±3.6	NR
Wenxia Wang (2016)	NR	NR	NR	NR	NR	NR	NR	NR	NR	22.5+3.9 18.8+2.9	NR	NR	NR
J. Slikkerveer (2016)	NR	NR	NR	NR	NR	51.8±15.2 51.5±16.5	164.4±49.2 168.5±54.4	81.9±40.2 86±41.4	NR	NR	NR	NR	NR
Gianluca Alunni (2017)	NR	2.53±0.68 1.39±0.52	NR	2.78±0.67 1.44±0.6	NR	NR	NR	NR	NR	21.1±9.6 14.05±10.05	13.4±9.28 9.5±9.48	NR	NR
Evgeny (2018)	NR	NR	NR	NR	NR	NR	NR	NR	NR	NR	NR	447.4±139.9 432.8±176.1	NR
Anderson S (2018)	NR	NR	NR	NR	NR	52.4±10.4 53.5±8.9	134±33 137±53	NR	5.4±0.7 5.3±0.7	15.33±8.60 16.60±8.06	NR	NR	NR
Massimo Slavich (2018)	NR	NR	NR	3.25±0.96 1.84±0.83	NR	58.75±4.99 60.68±3.23	90.50±11.85 97.37±22.32	NR	NR	NR	NR	358.21±135.01 303.25±109.59	NR
Yoku Kikuchi (2018)	384±91 435±122	NR	NR	NR	NR	56.3±14.7 58.8±12.8	NR	NR	NR	NR	NR	NR	11.1±6.3 8.9±10.7
Hong Yan CAI (2015)	360.69±116.79 434.15±86.29	1.85±0.21 1.23±0.08	67.58±13.03 77.54±10.84	1.85±0.15 1.19±0.08	1.00±0.27 0.50±0.19	NR	NR	NR	NR	NR	NR	NR	NR
Gitana Zuoziene (2011)	NR	NR	NR	3.2±0.41 2.2±0.41	NR	51.45±8.03 57.41±10.31	NR	NR	NR	NR	NR	NR	NR
C. Naber (2007)	NR	NR	NR	3.22±0.43 2.17±0.62	NR	NR	NR	NR	NR	NR	NR	NR	NR
A. Gutersohn (2003)	NR	NR	NR	3.3 ± 0.5 1.9 ±0.8	NR	NR	NR	NR	NR	NR	NR	NR	NR
Lothar Faber (2010)	NR	NR	NR	3.1±0.7 2.4+0.6	NR	NR	NR	NR	NR	NR	NR	NR	NR

CCS=Canadian Cardiology Society; LVEDD=left ventricular end-diastolic diameter; LVEDV=left ventricular end-diastolic volume; LVEF=left ventricular ejection fraction; LVESV=left ventricular end-systolic volume; 6MWT=six-min walking distance test; NR=no report; NYHA=New York Heart Association; SAQ=Seattle Angina Questionnaire; SDS=total difference score; SRS=total resting score; SSS=total load score

### Observing the Results of the Indicator Analyses

#### 6MWT

The number of studies included involving 6MWT were six^[4,15,19,22,28,29]^, with a total of 288 patients. The included study types were four single-arm studies, a cohort study, and one RCT. Heterogeneity results suggested that there was no heterogeneity in each study (*P*=0.45, I^2^=0%), and a meta-analysis was performed using a fixed effects model (Figure 3A). The results suggest that there is a significant difference between the CSWT and control groups regarding learning significance (MD 75.64, 95% CI 49.03, 102.25, *P*<0.00001). Subgroup analyses between the study types of the data were not statistically significant between the subgroups.

**Fig. 3A:**
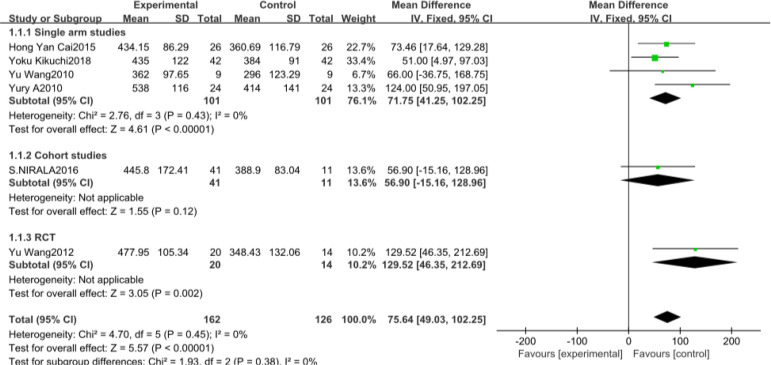
Forest map for the six-min walking distance test of the cardiac shock wave therapy group and the control group. CI=confidence interval; IV=inverse variance methods; RCT=randomised controlled trial; SD=standard deviation

#### NYHA

The number of studies included involving NYHA were seven^[13,15,19,20,24,28,29]^, with a total of 472 patients. The included study types were four single-arm studies and three cohort studies. Heterogeneity results suggested that there was heterogeneity in the statistics of each study (*P*<0.0001, I^2^=79%), and a meta-analysis was performed using a random analysis model (Figure 3B). The results suggest that there is a significant difference between the CSWT and control groups regarding learning significance (MD -0.70, 95% CI -0.92, -0.49, *P*<0.00001). Subgroup analyses between the study types of the data were not statistically significant between the subgroups.

**Fig. 3B:**
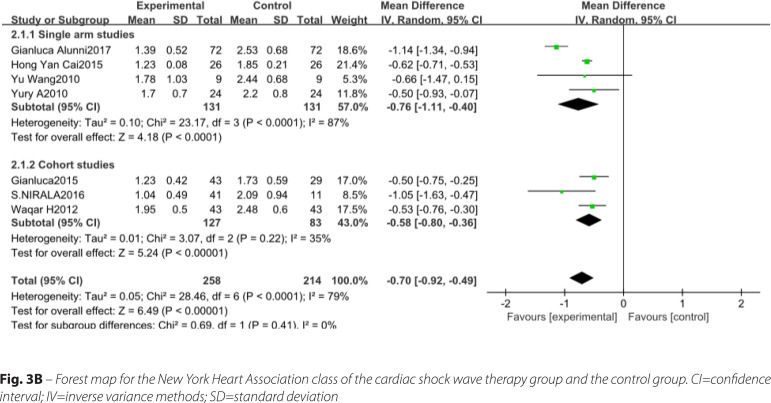
Forest map for the New York Heart Association class of the cardiac shock wave therapy group and the control group. CI=confidence interval; IV=inverse variance methods; SD=standard deviation

#### SAQ

The number of studies included involving SAQ were four^[15,19,22,28]^, with a total of 156 patients. The included study types were two single-arm studies, one cohort study, and one RCT. Heterogeneity results suggested that there was no heterogeneity in each study (*P*=0.13, I^2^=47%), and a meta-analysis was performed using a fixed effects model (Figure 3C). The results suggest that there is a significant difference between the CSWT and control groups regarding learning significance (MD 10.75, 95% CI 6.66, 14.83, *P*<0.00001). Subgroup analyses between the study types of the data were not statistically significant between the subgroups.

**Fig. 3C:**
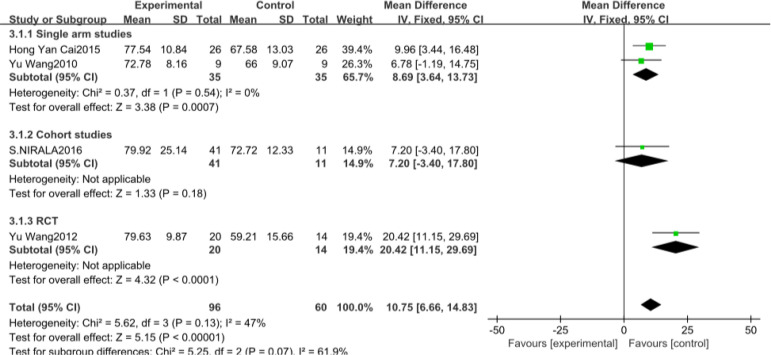
Forest map for the Seattle Angina Questionnaire of the cardiac short wave therapy group and the control group. CI=confidence interval; IV=inverse variance methods; RCT=randomised controlled trial; SD=standard deviation

#### CCS

The number of studies included involving CCS were 16^[11,13,15,16,19,20,24,27-34]^, with a total of 819 patients. The included study types were 12 single-arm studies and four cohort studies. Heterogeneity results suggested that there was heterogeneity in the statistical data of each study (*P*<0.00001, I^2^=88%), and a meta-analysis was performed using a random analysis model (Figure 3D). The results suggest that there is a significant difference between the CSWT and control groups regarding learning significance (MD -0.99, 95% CI -1.13, -0.84, *P*<0.00001). Subgroup analyses between the study types of the data were not statistically significant between the subgroups.

**Fig. 3D:**
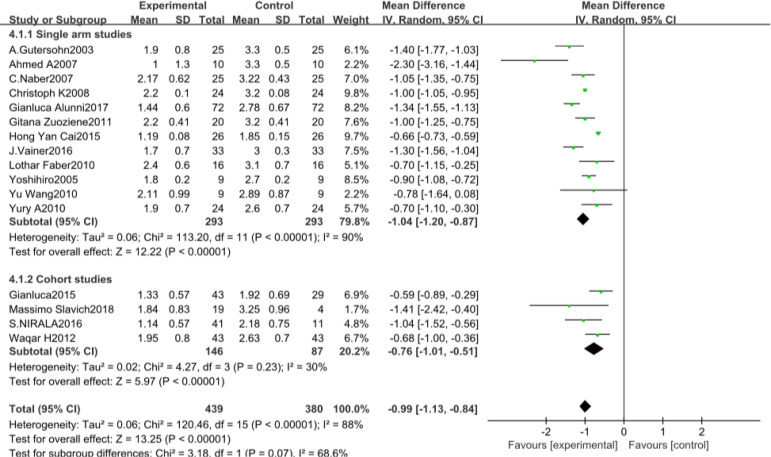
Forest map for the Canadian Cardiology Society class of the cardiac shock wave therapy group and the control group. CI=confidence interval; IV=inverse variance methods; SD=standard deviation

#### Nitrate Dosage

The number of studies included involving nitrate dosage were six^[6,15,16,19,28,33]^, with a total of 428 patients. The included study types were five single-arm studies and one cohort study. Heterogeneity results suggested that there was heterogeneity in the statistics of each study (*P*<0.00001, I^2^=94%), and a meta-analysis was performed using a random effect model. The results suggest that there is a significant difference between the CSWT and control groups regarding learning significance (MD -1.84, 95% CI -2.57, -1.12, *P*<0.00001). Subgroup analyses between the study types of the data were not statistically significant between the subgroups.

#### LVEF

The number of studies included involving LVEF were 12^[4,9,11,12,16-18,20,21,24,27,29]^, with a total of 566 patients. The included study types were nine single-arm studies, two cohort studies, and one RCT. Heterogeneity results suggested that there was no heterogeneity in each study (*P*=0.03, I^2^=49%), and a meta-analysis was performed using a fixed effects model. The results suggest that there is a significant difference between the CSWT and control groups regarding academic significance (MD 3.77, 95% CI 2.17, 5.37, *P*<0.00001). Subgroup analyses between the study types of data were performed, and statistical significance between the subgroups was found (*P*=0.01 < 0.05, I^2^=77.8%).

#### LVEDV

The number of studies included involving LVEDV were four^[9,11,17,18]^, with a total of 133 patients. The included study types were three single-arm studies and one cohort study. Heterogeneity results suggested that there was no heterogeneity in each study (*P*=1.00, I^2^=0%), and a meta-analysis was performed using a fixed effects model. The results suggest that there is no statistical relationship between the CSWT and control groups regarding academic significance (MD 5.51, 95% CI -4.85, 15.87, *P*=0.30). Subgroup analyses between the study types of the data were not statistically significant between the subgroups.

#### LVESV

The number of studies included involving LVESV were two^[17,18]^, with a total of 72 patients. These were single-arm studies. Heterogeneity results suggested that there was no heterogeneity in the statistics of each study (*P*=0.74, I^2^=0%), and a meta-analysis was performed using a fixed effect model. The results suggest that there is no statistical relationship between the CSWT and control groups regarding learning significance (MD 2.55, 95% CI -12.43, 17.53, *P*=0.54).

#### LVEDD

The number of studies included involving LVEDD were four^[9,12,18,21]^, with a total of 117 patients. The included study types were three single-arm studies and one cohort study. Heterogeneity results suggested that there was no heterogeneity in the study (*P*=0.25, I^2^=28%), and a meta-analysis was performed using a fixed effects model. The results suggest that there is a statistical relationship between the CSWT and control groups regarding learning significance (MD -0.41, 95% CI -0.78, -0.04, *P*=0.03). Subgroup analyses between the study types of the data were performed, and statistical significance between the subgroups was found (*P*=0.04, I^2^=75.1%).

#### SSS

The number of studies included involving SSS were seven^[6,9,13,14,20,29,33]^, with a total of 604 patients. The included study types were six single-arm studies and one cohort study. Heterogeneity results suggested that there was no heterogeneity in each study (*P*=0.15, I^2^=37%), and a meta-analysis was performed using a fixed effects model. The results suggest that there is a statistical difference between the CSWT and control groups regarding learning significance (MD -4.29, 95% CI -5.61, -2.96, *P*<0.00001). Subgroup analyses between the study types of the data were not statistically significant between the subgroups.

#### SRS

The number of studies included involving SRS were four^[6,13,20,29]^, with a total of 486 patients. The included study types were three single-arm studies and one cohort study. Heterogeneity results suggested that there was no heterogeneity in the study (*P*=0.71, I^2^=0%), and a meta-analysis was performed using a fixed effects model. The results suggest that there is a significant difference between the CSWT and control groups regarding learning significance (MD -2.90, 95% CI -4.85, -0.95, *P*=0.004). Subgroup analyses between the study types of the data were not statistically significant between the subgroups.

#### SDS

The number of studies included involving SDS were two^[4,6]^, with a total of 306 patients. These were single-arm studies. Heterogeneity results suggested that there was no heterogeneity in the study (*P*=0.87; I^2^=0%), and a meta-analysis was performed using a fixed effects model. The results suggest that there is no statistical relationship between the CSWT and control groups regarding learning significance (MD -1.99, 95% CI -4.73, 0.74, *P*=0.15).

#### Exercise Test

The number of studies included involving exercise test were five^[5,6,11,16,24]^, with a total of 484 patients. The included study types were two single-arm studies, two cohort studies, and one RCT. Heterogeneity results suggested that there was heterogeneity in the statistical data of each study (*P*=0.0004, I^2^=81%), and a meta-analysis was performed using a random effect model. Because the data units are not unified, the effect indicator selects the SMD mode. The results suggest that there was a statistically significant difference between the CSWT and control groups (SMD 0.57, 95% CI 0.12, 1.02, *P*=0.01). Subgroup analyses between the study types of the data were performed, and statistical significance between the subgroups was found (*P*=0.02, I^2^=75.3%).

### Sensitivity Analysis and Publishing Bias

For the sensitivity analysis of the heterogeneity index (NYHA, CCS, nitrate dosage, exercise test) in this study, the heterogeneity of the literature was removed one by one, and the heterogeneity did not change significantly after the indicators were removed. The design schemes, examination equipment, patient population, and statistical methods in each study did not exclude the indicators of a certain study. A funnel chart was drawn for each observation index, and the funnel chart indicated that the studies were generally symmetric and concentrated, so no indication was excluded (Figure 4). All models did not present obvious publication bias, calculated by the Egger’s test (*P*>0.05) (Figure 5).

**Fig. 4A:**
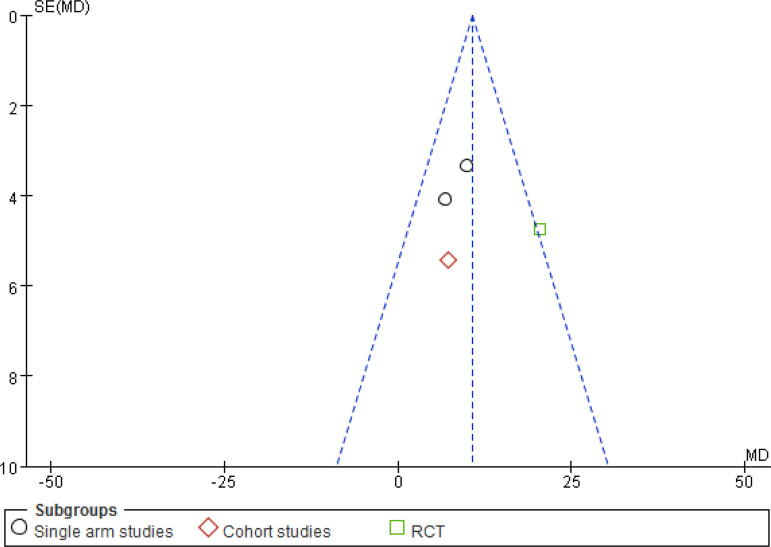
Funnel plot for the Seattle Angina Questionnaire of the cardiac shock wave therapy group and the control group. MD=mean difference; RCT=randomised controlled trial; SE=standard error

**Fig. 4B:**
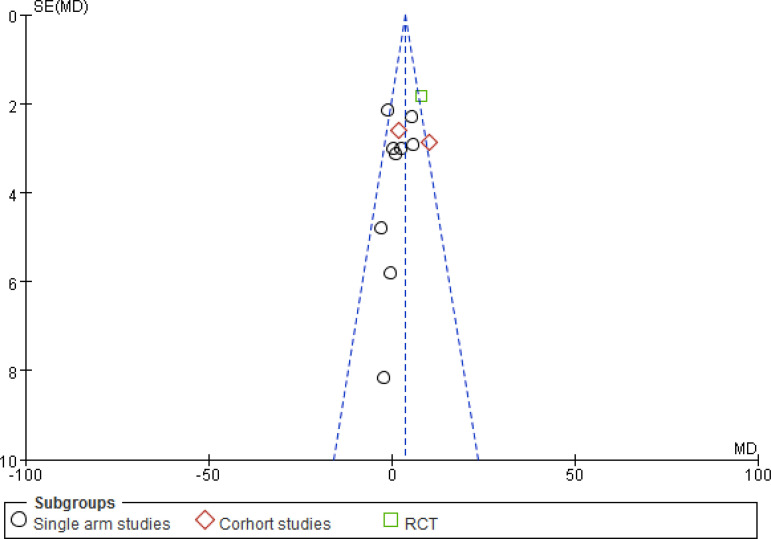
Funnel plot for the left ventricular ejection fraction of the cardiac shock wave therapy group and the control group. MD=mean difference; RCT=randomised controlled trial; SE=standard error

**Fig. 4C:**
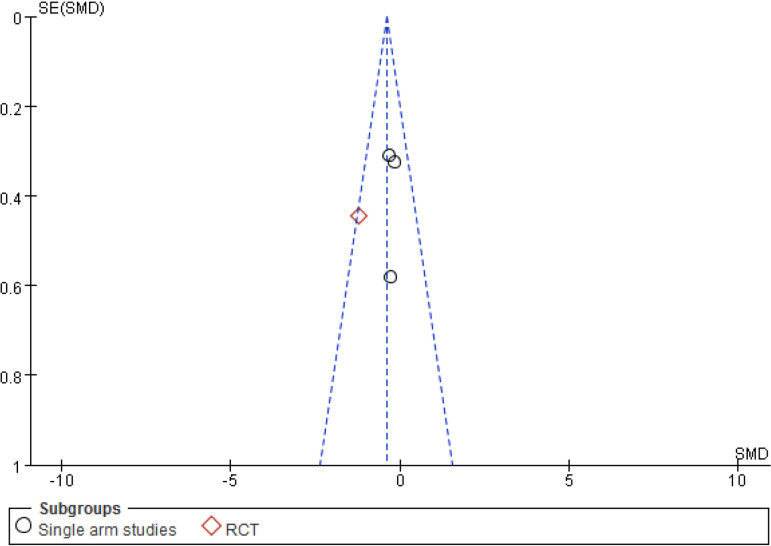
Funnel plot for the left ventricular end-diastolic diameter of the cardiac short wave therapy group and the control group. RCT=randomised controlled trial; SE=standard error; SMD=standard mean difference

**Fig. 4D:**
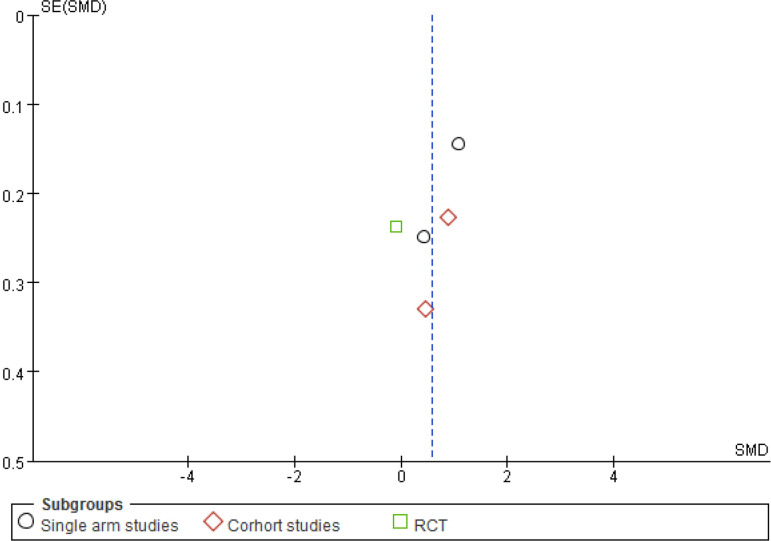
Funnel plot for the exercise test of the cardiac short wave therapy group and the control group. RCT=randomised controlled trial; SE=standard error; SMD=standard mean difference

**Fig. 5:**
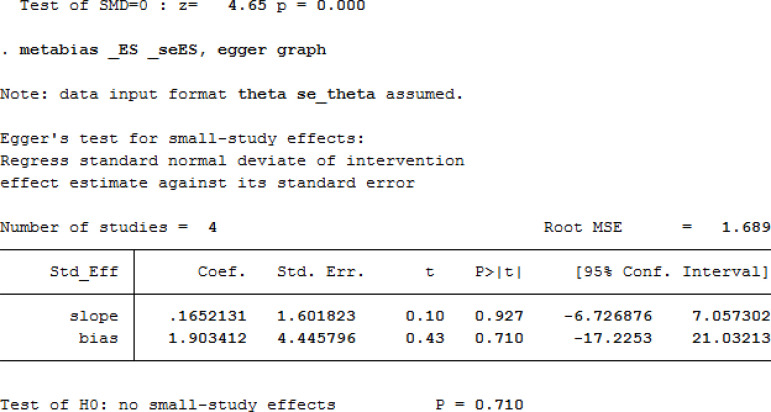
Egger’s test for the Seattle Angina Questionnaire of the cardiac short wave therapy group and the control group.

## DISCUSSION

Patients with coronary artery multivessel disease, who have lost PCI and CABG indications, often have large areas of myocardial necrosis, myocardial fibrosis, and decreased ventricular compliance. In this case, CSWT is a new approach in the treatment of CAD. The CSWT treatment system is a low-energy, high-voltage, high-frequency electromagnetic ultrasonic pulse that can generate huge pressure sound waves in an instant. The pulse wave is finely concentrated after interstitial reflection, and the myocardial ischaemic target area is accurately located by airborne real-time echocardiography. The surface electrocardiogram R wave triggers the extracorporeal shock wave to be released during the absolute refractory period of the electrocardiographic activity.

The titration release pulse pulsates energy to the target area, and the low-energy shock wave introduced into the myocardium *in vitro* generates mechanical shear stress, a cavitation effect, and ultrafine airflow in the myocardial tissue. Inward explosive force leads to changes in tissue subcellular structure, upregulation of vascular endothelial growth factor messenger ribonucleic acid and its receptor expression, stimulation of neovascularization, improvement of regional myocardial blood flow and capillary density^[35]^, production of anti-inflammatory factors, vasoactive activity substances (which softens tissue), increases penetration, improves blood circulation, promotes angiogenesis in the treatment target area and establishes collateral circulation, helps to increase blood supply to the heart, prevents ventricular remodelling, and improves myocardial ischaemia^[36]^. CSWT is used clinically, first to relieve the symptoms of refractory angina pectoris^[37]^. In addition to relieving symptoms of angina, CSWT treatment can also improve exercise tolerance and quality of life. The vast majority of clinical studies have shown that CSWT can improve patients with symptoms of angina, manifested as a decrease in CCS and an improvement in SAQ. This study used meta-analyses to evaluate the efficacy and safety of CSWT for the treatment of CAD, especially in the treatment of refractory CAD. A total of 31 articles were included, the data were summarized and extracted, and 26 articles were summarized. A total of 13 clinical observation indicators were analysed. The *P*-values of SAQ, CCS, nitrate dosage, LVEF, and SSS were all less than 0.05. This result indicates that CSWT treatment can increase the distance of 6MWT, which proves that CSWT can improve exercise tolerance; can improve CCS and reduce the dosage of nitrates, proving that CSWT can improve the frequency of angina pectoris; can improve SAQ and reduce NYHA classification, proving that CSWT can improve the patient's life treatment and body function; can improve LVEF and inhibit LVEDV and LVESV, proving that CSWT can inhibit ventricular remodelling; and can improve SSS and SRS, proving that CSWT can reduce the ischemic area, thereby reducing mortality. The therapeutic effect of CSWT and its up-regulation of NO in endothelial cells are synthetic related, improving endothelial function and promoting angiogenesis^[37]^.

In 2015, Wang Jing^[38]^ published a meta-analysis on CSWT, including 14 studies and involving a total of 516 patients. These studies have shown that CSWT can reduce the number of nitrates and improve the symptoms of angina and the CCS score. The angina is graded to improve cardiac function and improve left ventricular function. In 2017, Burneikaite et al.^[39]^ also published a meta-analysis on CSWT, including 39 studies and involving a total of 1,189 patients. All studies showed that CSWT could significantly improve angina symptoms and/or quality of life and improve exercise. Exercise tolerance, reduction in nitrate levels, and improvement in left ventricular function and myocardial perfusion were observed in most studies. The main indicators of this study are also consistent with the results of Wang Jing et al.^[38]^ and Burneikaite et al.^[39]^, which further confirms that CSWT can improve the symptoms of angina pectoris, exercise tolerance, and the quality of life of patients with refractory angina. In animal models and clinical trials of ischaemic cardiomyopathy with heart failure^[29]^, it has also been found that CSWT treatment can improve cardiac function in ischaemic cardiomyopathy and improve LVEF and NYHA cardiac function grading. In 2017, a Japanese scholar, Kagaya et al.^[40]^, observed, for the first time in clinical studies, that short-term CSWT treatment after emergency PCI for one week in 17 patients with acute myocardial infarction helped to improve LVEF further. This study further suggests that CSWT treatment can be performed earlier after acute myocardial infarction to improve the prognosis of these patients.

Negative indicators in this study, such as LVEDD and SDS, were included, and the number of patients was limited, which may be the main cause of negative results. Some of the observed indicators were heterogeneous in this study; although a subgroup analysis of the study type was performed, the source of heterogeneity could not be identified. Even if some indicators, such as LVEF and an exercise test, can reduce the heterogeneity after the study type subgroup, the heterogeneity in each subgroup of the relevant indicators still exists. This situation does not rule out the reasons why the sample size of each study is small, the data are too scattered, and the patient population is quite different. Therefore, the interpretation of the results of this study should be meticulous and cautious. In the evaluation of prognosis indicators for patients with CAD, 6MWT is a form of exercise test - with simple operation, good tolerance, and that can better reflect daily activities -, and this indicator has no heterogeneity in this study. Between the two groups, there are significant differences. Compared with 6MWT, SAQ is considered to be an important tool for assessing patients and has high reproducibility and sensitivity^[41,42]^. In this study, the SAQ index was low in heterogeneity and statistical significance, which can further affirm the therapeutic effect of CSWT.

CSWT treatment combines non-invasive treatment with improved microcirculation, reproducibility, technical feasibility, high safety, improvement in patients' heart function and patient's activity tolerance, reduction of drug use, and can promote the formation of cardiac collateral circulation. Improvement in myocardial perfusion can have broad prospects in the treatment of CAD.

The current clinical study of CSWT is mostly a self-control study before and after treatment in a single group of patients. Although this type of study is more feasible in a clinical setting, it cannot exclude the patient's own interference, and the sample size is small. Due to the clinical observation indicators are scattered and disunity is also one of the reasons for the heterogeneity of data, this is the shortcoming of this study. Prospective RCTs in the current study were small and were not able to perform the meta-analysis alone. In this study, a subgroup analysis of the study type was performed. In the subgroup, the three indicators LVEF, LVEDD, and exercise test were correlated with the type of study, but each of the three indicators showed statistical significance with CSWT. After sensitivity analysis of each study rejection, even statistical significance was more obvious.

### Limitations

All of the included articles are small in size. This data may include controversy, and it should be interpreted carefully as well. A rigorous meta-analysis should be accompanied with carefully balanced risk and benefit. It is easy to find some more studies with positive results about CSWT’s benefit, but this data from large randomized controlled studies are still limited. Some retrospective and cohort studies were conducted with rigour. If we abandon them, we may lose relatively precious data, especially for a newly developed treatment. Through lack of large RCTs in this study, it is difficult to avoid small unbalanced evaluation of the real effect. Obviously, it is essential to design the growth of sufficient powered, large, RCTs. Additional limitations include the short-term follow-up and a lack of standardization of outcome assessment methods.

## CONCLUSION

It can be concluded that CSWT has a significant improvement in the prognosis of patients with CAD. This conclusion needs further discussion before implementation and promotion in the clinic. The data sample size is small, and some of the data are skewed and can represent all of the data. CSWT is a relatively new method in the treatment of CAD, for the long-term efficacy of patients in symptom relief, cardiac function changes, and mortality, there is still a lack of corresponding data. It is worthwhile to carry out additional large-sample, multi-centre, different treatment programmes.

CSWT has the advantages of being non-invasive, painless, and safe. As an emerging non-pharmacological treatment, CSWT is effective in clinical research of CAD treatment and has a good safety record. It can be used with drug therapy or interventional therapy for coronary arteries. This bypass graft therapy provides a new treatment for severe CAD, especially angina pectoris and heart failure in advanced patients, and has created a new concept of CAD treatment. CSWT is mainly used in patients with chronic myocardial ischaemia and stable angina. In 2012, Russia and Germany extended the use of CSWT to the treatment of patients with ischaemic heart failure. Experts from various countries are still expanding the scope and application of CSWT treatment and expect to bring more benefits to patients. CSWT treatment will have broader application prospects in the future.

**Table:** 

Authors' roles & responsibilities	
HTY	Substantial contributions to the conception or design of the work; final approval of the version to be published
XX	Final approval of the version to be published
XGH	Substantial contributions to interpretation of data for the work; final approval of the version to be published
WJX	Substantial contributions to the acquisition and analysis of data for the work; final approval of the version to be published
TTW	Substantial contributions to the acquisition and analysis of data for the work; final approval of the version to be published
